# Low Concordance Between T-Cell Densities in Matched Primary Tumors and Liver Metastases in Microsatellite Stable Colorectal Cancer

**DOI:** 10.3389/fonc.2021.671629

**Published:** 2021-06-09

**Authors:** Vegar Johansen Dagenborg, Serena Elizabeth Marshall, Krzysztof Grzyb, Åsmund Avdem Fretland, Marius Lund-Iversen, Gunhild Mari Mælandsmo, Anne Hansen Ree, Bjørn Edwin, Sheraz Yaqub, Kjersti Flatmark

**Affiliations:** ^1^ Department of Tumor Biology, Oslo University Hospital, Oslo, Norway; ^2^ Institute of Clinical Medicine, University of Oslo, Oslo, Norway; ^3^ Department of Gastroenterological Surgery, Oslo University Hospital, Oslo, Norway; ^4^ Department of Pathology, Oslo University Hospital, Oslo, Norway; ^5^ Department of Hepato-Pancreato-Biliary Surgery, Oslo University Hospital, Oslo, Norway; ^6^ The Intervention Center, Oslo University Hospital, Oslo, Norway; ^7^ Institute of Medical Biology, The Arctic University of Norway-University of Tromsø, Tromsø, Norway; ^8^ Department of Oncology, Akershus University Hospital, Lørenskog, Norway

**Keywords:** colorectal cancer, colorectal liver metastases, cytotoxic T cell, helper T cells, regulatory T cells, T cell densities, tumor immune microenvironment

## Abstract

**Background:**

The subtype, density and location of tumor infiltrating T-cells are being explored as prognostic and predictive biomarkers in primary colorectal cancer (pCRC) and colorectal liver metastases (CLM). Very limited data exist comparing findings in pCRC and matched CLM.

**Patients and methods:**

Fifty-eight patients with available pCRC and matched CLM (57/58 microsatellite stable) were included in this OSLO-COMET substudy. In immunohistochemically stained sections, total (T_tot_), helper (TH), cytotoxic (CTL), and regulatory (Treg) T-cells were manually counted in hotspots from the invasive margin (IM), intratumor (IT), and tumor adjacent regions to determine T-cell densities.

**Results:**

A striking accumulation of T-cells was found in IM of both pCRC and CLM with much lower densities in the IT region, exemplified by T_tot_ of 2838 versus 340 cells/mm^2^, respectively, in CLM. The correlation at the individual level between T-cell densities in pCRC and corresponding CLM was poor for all regions and T-cell subtypes; for instance, the correlation coefficient (R^2^) for IM T_tot_ was 0.07. The IT TH : CTL and Treg : TH ratios were 2.94 and 0.44, respectively, in pCRC, and 1.84 and 0.24, respectively, in CLM.

**Conclusion:**

The observed accumulation of T-cells in the IM regions of pCRC and CLM with low penetration to the IT regions, combined with high TH : CTL and Treg : TH ratios, point to the presence of an immune suppressive microenvironment. T-cell densities of CLM differed markedly from the matched pCRC, indicating that to evaluate T-cell biomarkers in metastasis, the commonly available pCRC cannot serve as a surrogate for the metastatic tumor.

## Introduction

Colorectal cancer (CRC) is one of the most frequent cancers in the western world and about 50% of CRC patients will develop metastases during the course of disease, with the liver as the most frequent metastatic site (1, 2). Surgery represents a curative treatment option in approximately 25% of patients, but the majority of patients are not eligible for surgical interventions (3). Systemic chemotherapy now comprises a number of cytotoxic and targeted agents that are administered perioperatively, or as palliative treatment. Although the therapeutic opportunities have improved dramatically over the last two decades for patients with metastatic CRC (mCRC), the successes derived from introduction of immunotherapy have so far only been beneficial to microsatellite instable (MSI) cases, comprising approximately 5% of patients with mCRC (4). It is of high interest to understand the non-immunogenic cancers, such as microsatellite stable (MSS) CRC, to potentially identify opportunities to circumvent resistance and make immunotherapy a therapeutic option for the majority of mCRC patients (5). We recently showed up-regulation of immune-related genes and increased T-cell infiltration in colorectal liver metastases (CLM) after neoadjuvant chemotherapy, suggesting that cytotoxic therapies might favorably modify the tumor microenvironment to become more immunologically active (6, 7).

The subtype, density, and location of tumor infiltrating T-cells have been extensively investigated in primary CRC (pCRC), and strong associations with prognosis have been convincingly demonstrated (8–10), illustrating the importance of the adaptive immune system in CRC progression. Emerging evidence also suggests that patients with pCRC with a high density of T-cells benefit the most from adjuvant chemotherapy (11). In resected CLM, studies also point to an association between an abundance of infiltrating T-cells in the metastatic tumors and a favorable prognosis as well as response to chemotherapy (12–14). The prognostic value of infiltrating T-cells in pCRC for the outcome of subsequent CLM resection has been less extensively investigated and show conflicting results (14, 15).

Studies of T-cell infiltration in pCRC and matched CLM are heterogeneous with respect to cohort size, subtypes of T-cells analyzed, study design and research focus. Three studies have reported group comparisons of matched cases; one study showed concordance (n = 24) (16), while two studies (n = 16 and 69) (17, 18) made discordant findings when comparing pCRC and CLM. The only study where case-by-case correlation analyses were performed was recently published and showed poor correlation between the pCRC and CLM with respect to densities of CD3+ and CD8+ T-cells (n = 131) (14). Given the heterogeneity of existing data and the potential usefulness of T-cell quantification to portray the immune microenvironment in metastatic tumors, further studies are warranted in the field. In the present study, case-by-case and group analyses were performed to compare pCRC to matched CLM. Densities of CD3+, CD8+, CD4+, and FOXP3+ T-cells were analyzed using immunocytochemistry in a cohort of 58 CRC (of which 57 were MSS).

## Materials and methods

### Patients

Patients with resectable CLM were included in the *Oslo Randomized Laparoscopic Versus Open Liver Resection for Colorectal Metastases Study* (OSLO-COMET trial, NCT01516710) following informed consent (19). The study was approved by the Regional Committee for Health and Research Ethics in Norway (2011/1285/REK Sør-Øst B). All patients had primary adenocarcinoma in the colon or rectum. This OSLO-COMET sub study analyzed available samples from the first 71 patients included in the trial between February 2012 and April 2013. Thirteen cases were excluded from analysis because of unavailable or insufficient tumor content in the pCRC (n = 6) and CLM sample (n = 7), leaving 58 patients with pCRC tissue and corresponding CLM (bearing 84 metastases). In 53 of 58 pCRC and all CLM samples tumor adjacent tissue was evaluated. The pCRC was classified according to the TNM classification (International Union Against Cancer TNM classification 7th edition) (20). Performance status was classified using the Eastern Cooperative Oncology Group score (ECOG). The CLM clinical risk score (CRS) was calculated by giving 1 point for the following parameters: lymph node metastases in pCRC, <12 months from pCRC to diagnosis of CLM, multiple metastases, largest metastasis >5 cm, carcinoembryonic antigen (CEA) >200 µg/L. A CLM with CRS ≤ 2 was considered to have low risk of recurrence (21). For patients who had neoadjuvant chemotherapy prior to CLM resection, the response was classified using the principles of Response Evaluation Criteria in Solid Tumor 1.1 (RECIST 1.1) (22).

### Histological Assessment

Surgical specimens were formalin-fixed and paraffin embedded (FFPE) as part of routine pathology processing, and one representative tissue block was selected for analysis from each of the 58 patients (pCRC and corresponding CLM). Three regions were identified in hematoxylin and eosin (HE) stained sections for T-cell quantification: the tumor adjacent region in the colorectum and liver (N_Cr_ and N_Li,_ respectively); the intratumoural (IT) region and the invasive margin (IM). Selections were confirmed by a pathologist.

### T-Cell Densities Assessed by Immunohistochemistry (IHC)

Four-µm sections were deparaffinized and pretreated using PT-link, and subsequently stained using the Dako EnVision^™^ FLEX+ detection system (Agilent, California, USA). Serial sections were stained for the total number of T-cells (T_tot_ - CD3 rabbit monoclonal antibody; clone SP7; dilution 1:500, Thermo Scientific, California, USA), CTL (CD8 Novocastra^™^ lyophilized mouse monoclonal antibody; clone 4B11; 1:200, Leica Biosystems, Germany); helper T-cells (TH - CD4 mouse monoclonal; clone 4B12; ready-to-use; DAKO Denmark AS), and regulatory T-cells (Tregs - Anti-FOXP3 antibody; clone 236A/E7; dilution 1:400; Abcam, Cambridge, United Kingdom), all with positive controls. The slides were digitized and within scanned images multiple hotspots were selected: two representative areas from both N_Cr_ and N_Li_ tissue, three from the IT, and four from the IM region, where possible. The areas were marked on the HE slides, and the corresponding regions were located on serial sections stained for detection of T_tot_, CTL, TH and Treg. Due to poor quality staining, the following sections could not be evaluated: in N_Cr_ one sample of T_tot_ and one sample of CTL; in pCRC TH in both IM and IT in the same sample. T-cells were manually counted by investigator VJD who was blinded with respect to patient characteristics at the time of T-cell quantification. The T-cell density (cells/mm^2^) was calculated by dividing the sum of T-cells counted with the total area (mm^2^) selected within each region, using the Cell F software and an Olympus BX51 microscope with a ×10/0.30 FN26.5 lens connected to a XC30 camera. Immune cells were quantified over a median area (min – max) in IM of 1.15 mm^2^ (0.99–1.32) in pCRC and 1.14 mm^2^ (1.02–1.27) in CLM; in IT of 1.15 mm^2^ (1.13–1.16) in pCRC and 1.14 mm^2^ (1.07–1.22) in CLM; 0.76 mm^2^ (0.76–0.77) in N_Cr_ and 0.76 mm^2^ (0.76–0.76) for N_Li_. A total area of 1812 mm^2^ from all regions in both CRC and CLM and normal tissue was used for immune cell quantification. An area of 203 mm^2^ (11% of total area) from 26 tumor samples (10 pCRC and 16 CLM) was randomly selected from 16 patients for validation (by investigator SEM). Linear regression analyses comparing results by investigators VJD and SEM showed a high correlation (R^2^ > 0.95) with no significant differences comparing medians for each T-cell subtype.

### Calculation of Immune Score

For pCRC and CLM, an immune score of 0 to 4 was calculated using the method and cutoff values previously described by Galon et al. (8). One point each was given for T_tot_ over cutoff in IM (640 cells/mm^2^) and IT (300 cells/mm^2^), and 1 point each for CTL densities over cutoff in IM (370 cells/mm^2^) and IT (80 cells/mm^2^). An immune score of 0 to 2 was considered low and 3 to 4 high.

### Analysis of Microsatellite Instability (MSI)

The Idylla™ MSI Assay from Biocartis (Mechelen, Belgium) was used according to the manufacturer’s instructions to determine whether tumors were MSS or MSI (23). In 35 of the 58 cases, isolated DNA was available from CLM samples as previously described (7), while in 23 cases, FFPE tissue was used [from CLM resection sample (n = 20) or from the pCRC sample (n = 3)].

### Statistical Analysis

The variables were described using percentages and median with interquartile range (IQR) unless otherwise stated. Categorical variables were created for the following parameters by using medians as cutoff: age at pCRC resection, CEA levels at study CLM resection, and T-cell densities. Ratios were calculated for each tumor by dividing the following T-cell subtype densities: TH : CTL and Treg : TH. Paired variables were compared using Wilcoxon rank-sum test, independent variables by Mann-Whitney U. Correlations between T_tot_ and subtypes (CTL, TH, and Tregs) in pCRC and CLM, and N_Cr_ and N_Li_ were analyzed by linear regression. Results were reported with R^2^ values and *P*-values. Agreement between low or high immune score was assessed by Cohen’s kappa coefficient. For analyses related to outcome and immune score mean values were used for patients with multiple metastases, as we previously demonstrated that the variation in T-cell densities between metastases from the same patients was very low (6). Overall survival (OS) was calculated from pCRC resection to date of death (obtained from the Norwegian National Registry) or censor date (31st December 2017). Progression-free survival (PFS) was calculated from the time of study CLM resection to first event, last radiological follow-up, or death. Univariable analyses were performed by the Kaplan-Meier method to estimate OS and PFS. Reverse Kaplan-Meier method was used to estimate median follow-up. Hazard ratios (HR) were calculated using Cox proportional hazard analysis and were reported with 95% confidence interval (CI). A *P-*value < 0.05 was considered to be statistically significant. Data were collated and analyzed using IBM SPSS statistics software (version 25, IBM Corp, Armonk, NY).

## Results

### Clinicopathological Variables

The 58 patients from the OSLO-COMET trial sub study had the following clinicopathological features (**Table 1**): 32/58 (55%) patients were male, with a median age of 66 (60–72) at diagnosis of pCRC. The majority of patients had T3 or T4 pCRC (55/58; 95%), 34 patients (59%) had lymph node metastases in the pCRC specimen, and the tumors were located in the left colon or rectum in 42 cases (72%). Twenty-one cases (36%) had radiological evidence of metastatic disease at the time of pCRC diagnosis, and another 23 patients (40%) developed metastases within 12 months after surgery for pCRC. The median time from pCRC resection to diagnosis of CLM in the OSLO-COMET trial was 7 months (0–16). At the time of CLM resection, 42 patients (72%) were ECOG 0, 44 patients (76%) had a low CRS, and 28 patients (48%) had received neoadjuvant chemotherapy prior to CLM resection, where the majority had response or stable disease (24/28;86%). The median number of CLM was 1 (range, 1–6). Only one case (1.7%) was classified as MSI.

**Table 1:** Clinicopathological variables.

Variable			N (%)
Gender		Male	32 (55)
		Female	26 (45)
pCRC			
Age	Median (IQR)		66 (60–72)
TNM			
T		T1-T2	3 (5)
		T3	43 (74)
		T4	12 (21)
N		N0	24 (41)
		N1	19 (33)
		N2	15 (26)
M		M1	21 (36)
Histological grading		Low grade	51 (88)
		High grade	6 (10)
		Not classified	1 (2)
Anatomical location		Right side	16 (28)
		Left side/rectum	42 (72)
Immune score	Low	0	3 (5)
		1	4 (7)
		2	12 (21)
	High	3	13 (22)
		4	26 (45)
CLM			
Performance status	ECOG	0	42 (72)
		1–2	16 (28)
Clinical risk score	Low	0	7 (12)
		1	18 (31)
		2	19 (33)
	High	3	13 (22)
		4	1 (2)
		5	0
Number of CLM	Median (Range)		1 (1–6)
Cases with multiple metastases			19 (33)
CEA CLM resection	Median (Range)		4 (1–233)
NACT			28 (48)
Response (RECIST 1.1)		Partial response	14 (50)
		Stable disease	10 (36)
		Progressive disease	4 (14)

pCRC, primary colorectal cancer; T, tumor; N, lymph node metastases; M, distant metastases; CLM, colorectal liver metastases; ECOG, The Eastern Cooperative Oncology Group performance status; CEA, carcinoembryonic antigen; NACT, neoadjuvant chemotherapy; RECIST, response evaluation criteria in solid tumors.

### The Highest T-Cell Densities Were Detected in IM CLM

**Figure 1:**
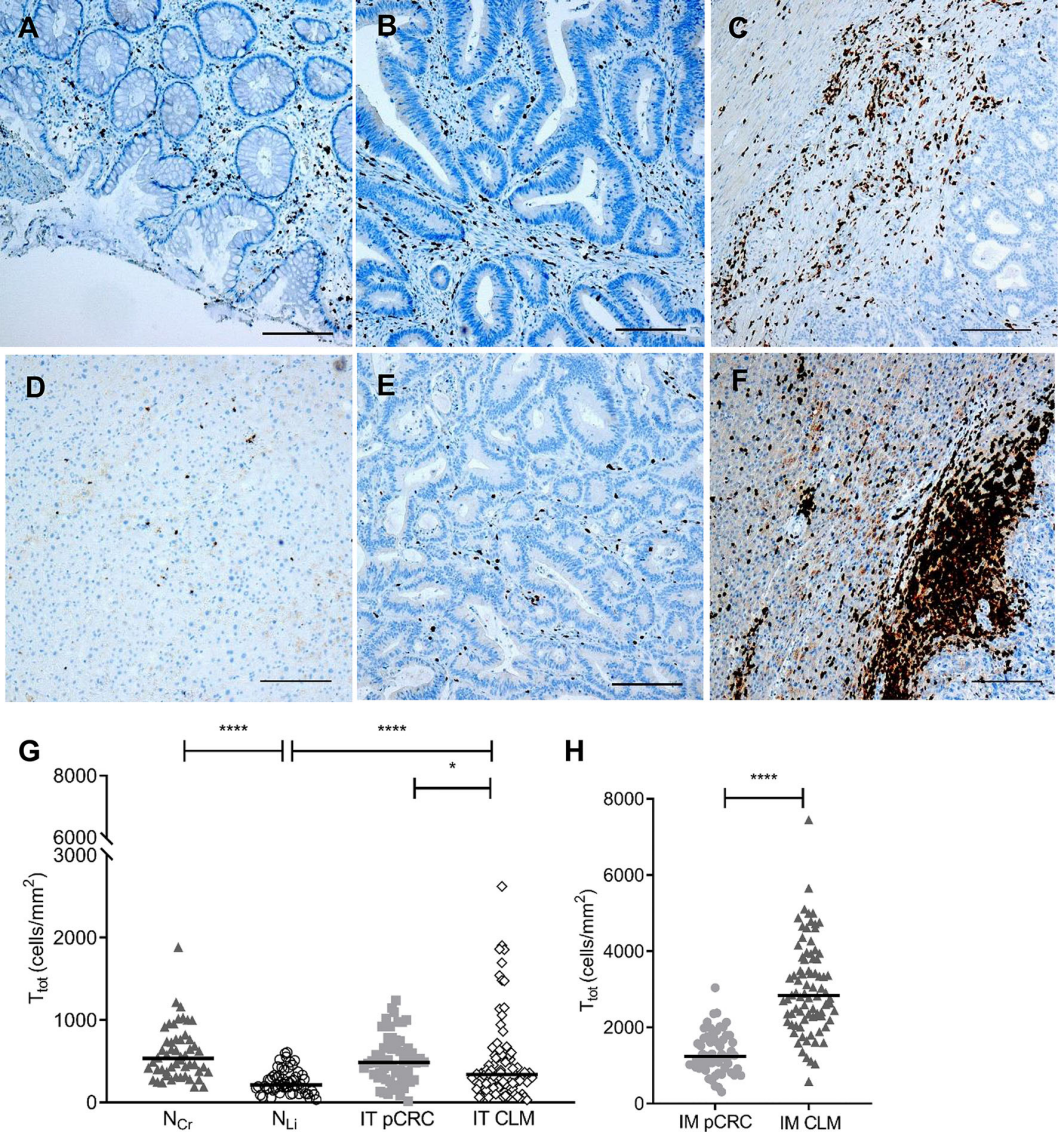
Total T-cell densities in in tumor adjacent colorectum (N_Cr_) and liver (N_Li_), intratumor (IT) and invasive margin (IM) in primary colorectal cancer (pCRC) and colorectal liver metastases (CLM). Representative immunohistochemistry images show T-cell densities (T_tot_; CD3+). Images were acquired at 4× magnification and the black line represents 0.2 mm. The following regions are shown: **(A)** N_Cr_; **(B)** IT pCRC; **(C)** IM pCRC; **(D)** N_Li_; **(E)** IT CLM; **(F)** IM CLM. **(G, H)** show dot density plots comparing pairwise total T-cell densities (T_tot_) in the same regions. The black lines represent median values. Only significant differences are shown. *P-value < 0.05; ****P-value < 0.0001.

Examining the tumor adjacent tissues, the T_tot_ density in N_Cr_ was double that of N_Li_ (533 cells/mm^2^ (371–764) versus 221 cells/mm^2^ (122–319); *P*-value < 0.0001) (**Figure 1**, **Table 2**). The T_tot_ densities of IT and N_Cr_ in pCRC were similar (*P*-value = 0.4), comparatively, T_tot_ density of IT CLM was significantly higher than N_Li_ (340 cells/mm^2^ (184–569) versus 221 cells/mm2 (122–319); *P*-value < 0.0001). The highest density of T-cells was found in IM of both pCRC and CLM, where IM CLM had more than twice the density found in IM pCRC (2838 cells/mm^2^ (2292–3841) versus 1244 cells/mm^2^ (933–1749); *P*-value <0.0001) (**Figure 1**). T_tot_ density in IM CLM was 13-fold that quantified in N_Li_.

**Table 2:** Median T-cell densities (cells/mm^2^) in tumor adjacent tissue, IT and IM in pCRC and CLM.

	N_Cr_	pCRC		N_LI_	CLM	
		IT	IM		IT	IM
T_tot_	533 (371–764)	485 (284–706)	1244 (933–1749)	221 (122–319)	340 (184–569)	2838 (2292–3841)
CTL	232 (146–357)	106 (51–196)	497 (315–720)	121 (68–213)	101 (49–222)	1084 (763–1423)
TH	287 (188–422)	353 (191–481)	772 (614–1004)	77 (51–123)	199 (115–397)	1904 (1454–2715)
Tregs	58 (41–103)	144 (74–224)	223 (163–360)	3 (0–5)	43 (21–85)	229 (123–377)

pCRC, primary colorectal cancer; CLM, colorectal liver metastases; N_Cr_, tumor adjacent colon and rectum; N_LI_, tumor adjacent liver tissue; IT, intratumor; IM, invasive margin; T_tot_, total amount of T-cells (CD3+), CTL, cytotoxic T-cells (CD8+); TH, helper T-cells (CD4+); Tregs, regulatory T-cells (FOXP3+).

### TH Was the Dominating T-Cell Subtype in Both pCRC and CLM

**Figure 2:**
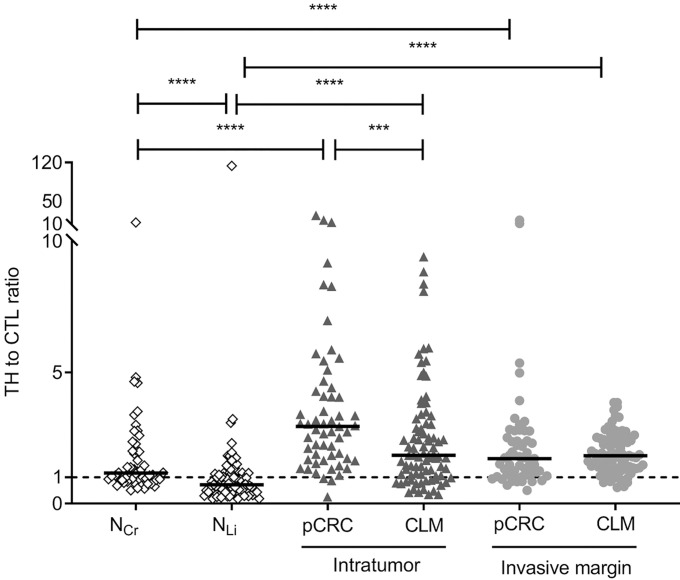
Dot density plots showing ratios of helper T-cells to cytotoxic T-cells (TH : CTL) in tumor adjacent colorectum (N_Cr_) and liver (N_Li_), intratumor (IT) and invasive margin (IM) in primary colorectal cancer (pCRC) and colorectal liver metastases (CLM). The black lines represent median values. The dashed line indicates a ratio of 1.0. Regions are compared pairwise using the Mann-Whitney U test and only significant differences are shown. ***P-value < 0.001; ****P-value < 0.0001.

The densities for the CTL and TH subtypes mirrored the T_tot_ densities for all regions, with the highest CTL and TH densities found in IM CLM followed by IM pCRC (**Figure 1**, **Table 2**). TH was the dominating T-cell subtype in the tumor regions (IT and IM) of both pCRC and CLM, with a median TH : CTL ratio of more than one (**Figure 2** and **Supplementary Table 1**). The highest TH : CTL ratio was observed in IT pCRC (2.94 (1.70–4.35) vs 1.84 (1.07–3.04)) in IT CLM, *P*-value < 0.001. Tumor adjacent tissue had TH : CTL ratios close to or below 1 (1.16 (0.89–2.03) in N_Cr_ and 0.72 (0.44–1.14) in N_Li_). The TH : CTL ratio (**Figure 2**) observed in IM pCRC and IM CLM were similar (1.71 (1.09–2.39) vs 1.83 (1.36–2.50), *P*-value = 0.4) (**Supplementary Tables 1**, **2**).

### The Highest Treg/TH Ratios Were Observed in IT pCRC

**Figure 3:**
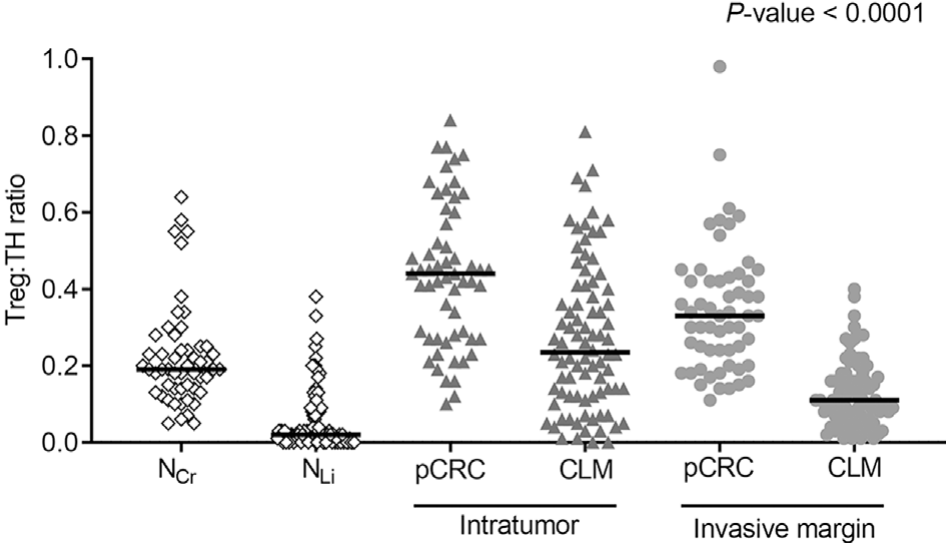
Dot density plots showing ratios of regulatory T-cells to helper T-cells (Treg : TH) in tumor adjacent colorectum (N_Cr_) and liver (N_Li_), intratumor (IT) and invasive margin (IM) in primary colorectal cancer (pCRC) and colorectal liver metastases (CLM). Regions were compared pairwise using the Mann-Whitney U test/Wilcoxon signed rank tests. Pairwise comparisons were all significant with a *P*-value < 0.0001 (**Supplementary Tables 1**, **2**).

The densities of the Treg subtype were generally low in all analyzed regions compared to CTL and TH (**Table 2**). Numerically, the densities were higher in pCRC and N_Cr_ than in CLM and N_Li_, with exception of the IM region where the Treg densities were numerically similar in pCRC and CLM (**Table 2**). The Treg : TH ratios were higher in the pCRC compared to corresponding CLM regions (**Figure 3** and Supplementary **Table 2**). The highest Treg : TH ratio was observed in IT pCRC (0.44 (0.27–0.59)), nearly double that found in IT CLM (0.24 (0.12–0.41), *P*-value < 0.0001). In IM pCRC the Treg : TH ratio was 3 times higher than in IM CLM (0.33 (0.22–0.43) vs 0.11 (0.07–0.18); *P*-value < 0.0001) (**Table 2**). Similar observations were made in the tumor adjacent regions, where Treg : TH in N_Cr_ was almost 10 times higher than in N_Li_ 0.19 (0.14–0.25) vs 0.02 (0.00–0.08); *P*-value < 0.0001).

### No Correlation Between T-Cell Densities in pCRC and Matched CLM

**Figure 4:**
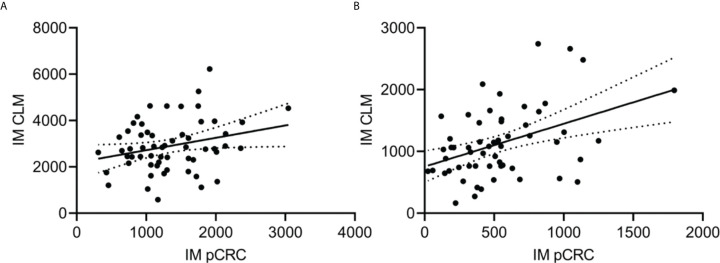
Representative scatter plots investigating correlation between T-cell densities (cells/mm^2^) in the invasive margin (IM) of primary colorectal cancer (pCRC; x-axis) and colorectal liver metastases (CLM; y-axis). The black line represents the regression line with a 95% confidence interval. **(A)** Total T-cell densities in IM, correlation coefficient (R^2^) = 0.07. **(B)** Cytotoxic T-cell densities in IM, R^2^ = 0.18.

The correlation between T-cell densities in pCRC and corresponding CLM was poor for all regions, exemplified by IM T_tot_ pCRC and CLM, R^2^ = 0.07 (**Figure 4**, **Supplementary Table 3**). Thirty-nine of 58 (67%) pCRC samples were scored to have a high immune score (**Table 1**). When the same cutoff values were applied to CLM, 34/58 (59%) were found to have a high immune score. However, only 50% of pCRC and corresponding CLM had a concordant score (i.e. high/high or low/low; Cohen’s kappa coefficient = −0.6).

### Associations Between Clinicopathological Variables and Long-Term Outcome

The follow-up time after pCRC resection was an estimated median of 79 months (95% CI: 67–91) and an estimated median of 64 months (95% CI: 61–96) after CLM study resection. Twenty-six (45%) patients died during follow-up. After CLM resection the estimated 5-year OS was 58%, and median OS was not reached. The median PFS was 25 months (95% CI: 10–40) with a median follow-up time of 60 months (95% CI: 54–67). Forty-one (71%) patients had an event after CLM resection (liver metastases (n=16); lung metastases (n=7); recurrence other sites than liver and lung (n=5); recurrence at multiple sites (n=8); other cancer (n=4); death without cancer recurrence (n=1)). Variables associated with OS and PFS in this cohort were similar to previously published results from the OSLO-COMET trial (7), and there were no associations between T-cell densities or immune score and long-term outcome (**Supplementary Table 4**).

## Discussion

In this ancillary study from the OSLO-COMET trial, T-cell densities (T_tot_, CTL, TH and Tregs) were quantified using IHC in matched pCRC and CLM samples from 58 patients with resectable CLM, of which only one was classified as MSI. The low MSI frequency is well in line with the majority (72%) of the primary tumors being located in the left colon, where MSI is typically much less common than in right-sides cases (2% *vs* 17%) respectively (24). This is also in accordance with previous molecular findings in a subset of this cohort showing low frequency of *BRAF* mutated cases and a dominance of the CMS2 subtype (7). A high concordance was previously demonstrated between the primary tumor and corresponding metastases for the most common driver mutations and MSI status in CRC, suggesting that the primary site adequately reflects the individual patient’s disease with respect to these parameters (25, 26). In contrast, T-cell densities in pCRC and matched CLM have been investigated in a very limited number of cases. Even when matched data have been available, results have either been conflicting (14, 16), or the focus has remained on reporting group comparisons (18). In our cohort, the correlation between pCRC and CLM from the same individual was extremely low for total T-cell densities, and for the investigated T-cell subtypes, and consequently also for the calculated immune score. This could possibly be related to the distinct differences in T-cell densities and T-cell composition in N_Cr_ and N_Li_ visualizing a difference in immune function in the colorectum compared to the liver. The pCRC immune contexture therefore does not seem to be predictive for CLM, which should be kept in mind as tumor infiltrating T-cells are emerging as a potential predictive biomarker for response to immune therapy.

A striking accumulation of T-cells in the IM regions was observed, particularly in CLM, contrasting the exceptionally low T-cell densities detected in N_Li_. The findings are in accordance with previous reports and likely represent recruitment of T-cells to the tumor site in both the colorectum and liver (12, 13, 18). The prominent differences in T-cell densities between the IM and IT regions demonstrate an inefficiency of penetration into the tumor regions, which could be influenced by a number of factors, such as immunosuppressive signals in the extracellular matrix, lack of T-cell activation by antigen presenting cells, and the presence of immunosuppressive immune cells and chemokines (27, 28). The median IT CTL density of our pCRC samples was very similar to the CTL density previously reported for MSS pCRC tumors (106 cells/mm^3^ *vs* ~100 cells/mm^3^, respectively) (29), which is in line with all our cases, except one, being MSS. Interestingly, the IT CTL densities of pCRC and CLM were similar in our study, and only half of the CTL density found in MSI pCRC (29), suggesting that the immunosuppressed and immune excluded features of MSS pCRC were preserved in CLM (5). We previously demonstrated a transient increase in T-cell densities in CLM after neoadjuvant chemotherapy and also that differentially expressed genes related to “Dendritic Cell Maturation” and “Cellular Movement, Immune Cell Trafficking” were up-regulated in the patients who had neoadjuvant chemotherapy compared to patients that had no chemotherapy in the OSLO-COMET cohort (6, 7). Based on these results, we have initiated the ongoing “Colorectal Cancer METastasis - Shaping Anti-tumor IMMunity by Oxaliplatin” (METIMMOX) trial (NCT03388190), where cytotoxic chemotherapy is being studied as a potential means to overcome immune suppression in MSS mCRC.

The highest TH : CTL ratios were found in the IT region of pCRC, followed by the IT of CLM and IM of pCRC/CLM., contrasting ratios close to 1 in the adjacent tumor tissues. A high TH : CTL ratio has been associated with a metastatic phenotype in pCRC and with poor prognosis in both pCRC and CLM (30–32). The interplay between T-cell subtypes has been recognized as important in controlling tumor growth, with TH subsets orchestrating adaptive immune responses and CTL exerting classic anti-tumor effects (33, 34). The immunosuppressive Treg subtype of TH cells (35) exhibited the lowest density of the investigated T-cell subtypes in most regions, but the relative proportion of Tregs to TH was highly variable. In the N_Cr_ Tregs constituted about 20% of TH, which is in agreement with previous reports, showing that Tregs are quite abundant in the colorectum (36). In the IT pCRC region the Treg: TH ratio was particularly high, with Tregs constituting 44% of TH cells. The immune inhibitory effect of Tregs on CTL and TH is exerted by several mechanisms, for instance by expression of immune check-point proteins, such as cytotoxic T-lymphocyte–associated antigen 4 and programmed death-ligand 1 (PD-L1), in addition to secreting immunosuppressive cytokines, such as interleukin-10, prostaglandin E2, and transforming growth factor β (37, 38). Interestingly, PD-L1 was expressed in only 1/92 CLM cases from the OSLO-COMET trial (including all the CLM cases in this study) (6). The high tumor TH : CTL and Treg : TH ratios are in line with all the investigated cases having developed metastatic disease, and underline the role of TH in the immune suppressive tumor microenvironment of pCRC and CLM. The interesting findings derived from these analyses, particularly regarding T-cell subsets, underline the limitation related to use of only four T-cell markers. Efforts to further characterize the TH subsets and other immune cells, such as macrophages and neutrophils, in CLM are warranted. For such analyses more high-throughput methods should be employed.

Using the immune score classification developed by Galon et al. (8), the majority (67%) of pCRC in our cohort had a favorable immune score. This finding is somewhat unexpected, since the entire cohort was composed of pCRC cases that developed CLM (76% within the first year of pCRC surgery), and in contrast to previous reports where a high immune score in pCRC was associated with a low disease recurrence rate (8, 39). The lack of agreement could reflect an intended “selection bias” based on clinical assessment of CLM resectability in the OSLO-COMET trial, resulting in differences between the pCRC cohorts. When the immune score criteria were applied to the metastatic tumors, 59% of the CLM were classified in the high immune score group. However, as previously noted, concordance between individual immune score in pCRC and matched CLM was poor and there were no associations between CLM T-cell subtype densities or calculated immune score and long-term outcome. In contrast to our results, some studies have demonstrated associations between a high CLM immune score and a favorable outcome after CLM resection (12–14). Regretfully, although our cohort was sizable in the context of analyzing the immune microenvironment, it was not planned or powered for detection of small differences in outcome. Taken together, there seems to be a need for further studies to clarify the utility of CLM T-cell density assessment as a tool for predicting prognosis after metastasis surgery. If the role of T-cell quantification could be established in this context, it could be a useful asset for treatment selection in CLM patients.

In this study of matched pCRC and CLM samples, accumulation of T-cells in the IM regions with low penetration to the IT regions combined with high TH : CTL and Treg : TH ratios pointed to an immune suppressive microenvironment. With the observed low correlation between tumor T-cell infiltration in pCRC and matched CLM, analysis of T-cells in pCRC is clearly not a valid biomarker for CLM. This is of particular importance in non-resectable CLM, where resection specimens will not be available. For the non-resectable setting, establishment of methods that require less material for assessment of T-cell infiltration is warranted, using for instance sequencing based approaches. Patient cohorts with available resection specimens, such as ours, may be useful for validation of novel analytical approaches.

## Data Availability Statement

The raw data supporting the conclusions of this article will be made available by the authors, without undue reservation.

## Ethics Statement

The studies involving human participants were reviewed and approved by the Regional Committee for Health and Research Ethics in Norway (2011/1285/REK SørØst B). The patients/participants provided their written informed consent to participate in this study.

## Author Contributions

VD: collected and analyzed data, and wrote manuscript. SM: analyzed and validated data, and reviewed the manuscript. ÅF: included patients and collected data, and reviewed the manuscript. KG and ML-I: analyzed histological slides and reviewed the manuscript. GM and AR: project idea, reviewed manuscript, and organized funding. SY and KF: project idea, project planning, co-wrote manuscript, and organized funding. All authors contributed to the article and approved the submitted version.

## Funding

VD was supported by the Norwegian Research Council; grant 218325 (to the project: Actionable Targets in Cancer Metastasis—From Bed to Bench to Byte to Bedside project) and the Radium Hospital Foundation. SM was supported by Norwegian Cancer Society; grant 182496 (to the project: Colorectal Cancer METastasis - Shaping Anti-tumor IMMunity by Oxaliplatin).

## Conflict of Interest

The authors declare that the research was conducted in the absence of any commercial or financial relationships that could be construed as a potential conflict of interest.
