# Supplementary material for: Low Concordance Between T-Cell Densities in Matched Primary Tumors and Liver Metastases in Microsatellite Stable Colorectal Cancer
Source: Front Oncol. 2021 Jun 9;11:671629. doi: 10.3389/fonc.2021.671629 (PMC8220067; doi:10.3389/fonc.2021.671629)
Supplement: Supplementary file 1 [file Table_1.docx]

Supplementary material

| Supplementary Table 1. T-cell density (cells/mm^2^) and ratios comparing pCRC vs CLM   \|  \|  \| N_Cr_/pCRC \| N_Li_/CLM \| *P* \| \| --- \| --- \| --- \| --- \| --- \| \| IM \| T_tot_ \| 1244 (933 - 1749) \| 2838 (2292 - 3841) \| <0.0001 \| \|  \| CTL \| 497 (315 - 720) \| 1084 (763 - 1423) \| <0.0001 \| \|  \| TH \| 772 (614 - 1004) \| 1904 (1454 - 2715) \| <0.0001 \| \|  \| Treg \| 223 (163 - 360) \| 229 (123 - 377) \| 0.386 \| \| IT \| T_tot_ \| 485 (284 - 706) \| 340 (184 - 569) \| 0.031 \| \|  \| CTL \| 106 (51 - 196) \| 101 (49 - 222) \| 0.771 \| \|  \| TH \| 353 (191 - 481) \| 199 (115 - 397) \| 0.006 \| \|  \| Treg \| 144 (74 - 224) \| 43 (21 - 85) \| <0.0001 \| \| N \| T_tot_ \| 533 (371 - 764) \| 221 (122 - 319) \| <0.0001 \| \|  \| CTL \| 232 (146 - 357) \| 121 (68 - 213) \| <0.0001 \| \|  \| TH \| 287 (188 - 422) \| 77 (51 - 123) \| <0.0001 \| \|  \| Treg \| 58 (41 - 103) \| 3 (0 - 5) \| <0.0001 \| \| IM \| TH:CTL \| 1.71 (1.09 - 2.39) \| 1.83 (1.36 - 2.50) \| 0.374 \| \|  \| Treg:TH \| 0.33 (0.22 - 0.43) \| 0.11 (0.07 - 0.18) \| <0.0001 \| \| IT \| TH:CTL \| 2.94 (1.70 - 4.35) \| 1.84 (1.07 - 3.04) \| <0.001 \| \|  \| Treg:TH \| 0.44 (0.27 - 0.59) \| 0.24 (0.12 - 0.41) \| <0.0001 \| \| N \| TH:CTL \| 1.16 (0.89 - 2.03) \| 0.72 (0.44 - 1.14) \| <0.0001 \| \|  \| Treg:TH \| 0.19 (0.14 - 0.25) \| 0.02 (0.00 - 0.08) \| <0.0001 \|   Pairwise comparisons using Mann-Whitney U test. *P* < 0.05 was considered as statistically significant.  N, tumor adjacent colorectum (N_Cr_) and tumor adjacent liver (N_Li_); IT, intratumor; IM, invasive margin; pCRC, primary colorectal cancer; CLM, colorectal liver metastasis; T_tot_, total amount of T-cells, CTL, cytotoxic T-cells, TH, Helper T-cells; Treg, regulatory T-cells  Supplementary Table 2. T-cell densities (cells/mm^2^) and ratios compared between regions in pCRC and in CLM   \|  \|  \| N_Cr_ \| *P* \| IT \| *P* \| IM \| *P* \| N_Cr_ \| \| --- \| --- \| --- \| --- \| --- \| --- \| --- \| --- \| --- \| \| pCRC \| T_tot_ \| 533 (371 - 764) \| 0.4 \| 485 (284 - 706) \| <0.0001 \| 1244 (933 - 1749) \| <0.0001 \| 533 (371 - 764) \| \| CTL \| 232 (146 - 357) \| <0.0001 \| 106 (51 - 196) \| <0.0001 \| 497 (315 - 720) \| <0.0001 \| 232 (146 - 357) \| \| TH \| 287 (188 - 422) \| 0.3 \| 353 (191 - 481) \| <0.0001 \| 772 (614 - 1004) \| <0.0001 \| 287 (188 - 422) \| \| Treg \| 58 (41 - 103) \| <0.0001 \| 144 (74 - 224) \| <0.0001 \| 223 (163 - 360) \| <0.0001 \| 58 (41 - 103) \| \| TH:CTL \| 1.16 (0.89 - 2.03) \| <0.0001 \| 2.94 (1.70 - 4.35) \| <0.0001 \| 1.71 (1.09 - 2.39) \| 0.06 \| 1.16 (0.89 - 2.03) \| \| Treg:TH \| 0.19 (0.14 - 0.25) \| <0.0001 \| 0.44 (0.27 - 0.59) \| <0.0001 \| 0.33 (0.22 - 0.43) \| <0.0001 \| 0.19 (0.14 - 0.25) \| \|  \|  \|  \|  \|  \|  \|  \|  \|  \| \|  \|  \| N_Li_ \|  \| IT \|  \| IM \|  \| N_Li_ \| \| CLM \| T_tot_ \| 221 (123 - 319) \| <0.0001 \| 340 (184 - 569) \| <0.0001 \| 2838 (2292 - 3841) \| <0.0001 \| 221 (122 - 319) \| \| CTL \| 121 (68 - 213) \| 0.5 \| 101 (49 - 222) \| <0.0001 \| 1084 (763 - 1423) \| <0.0001 \| 121 (68 - 213) \| \| TH \| 77 (51 - 123) \| <0.0001 \| 199 (115 - 397) \| <0.0001 \| 1904 (1454 - 2715) \| <0.0001 \| 77 (51 - 123) \| \| Treg \| 3 (0 - 5) \| <0.0001 \| 43 (21 - 85) \| <0.0001 \| 229 (123 - 377) \| <0.0001 \| 3 (0 - 5) \| \| TH:CTL \| 0.72 (0.44 - 1.14) \| <0.0001 \| 1.83 (1.36 - 2.50) \| 0.1 \| 1.84 (1.07 - 3.04) \| <0.0001 \| 0.72 (0.44 - 1.14) \| \| Treg:TH \| 0.02 (0.00 - 0.08) \| <0.0001 \| 0.11 (0.07 - 0.18) \| <0.0001 \| 0.24 (0.12 - 0.41) \| <0.0001 \| 0.02 (0.00 - 0.08) \|   Pairwise comparisons using Wilcoxon rank-sum test. *P* < 0.05 was considered as statistically significant.  pCRC, primary colorectal cancer; CLM, colorectal liver metastasis; N_Cr_, tumor adjacent colorectum; N_Li_, tumor adjacent liver; IT, intratumor; IM, invasive margin; T_tot_, total amount of T-cells, CTL, cytotoxic T-cells, TH, Helper T-cells; Treg, regulatory T-cells  Supplementary Table 3. Linear correlation between total T-cells and subtypes in IT, IM and adjacent normal tissues in colon/rectum and liver.   \| Colon and rectum versus liver \| R^2^ \| *P* \| \| --- \| --- \| --- \| \| T_tot_ IM \| 0.07 \| 0.04 \| \| CTL IM \| 0.18 \| 0.00 \| \| TH IM \| 0.05 \| 0.08 \| \| Treg IM \| 0.05 \| 0.08 \| \| T_tot_ IT \| 0.01 \| 0.44 \| \| CTL IT \| 0.12 \| 0.01 \| \| TH IT \| <0.01 \| 0.87 \| \| Treg IT \| <0.01 \| 0.79 \| \| T_tot_ N_Cr_ / N_Li_ \| 0.01 \| 0.54 \| \| CTL N_Cr_ / N_Li_ \| <0.01 \| 0.99 \| \| TH N_Cr_ / N_Li_ \| 0.01 \| 0.57 \| \| Treg N_Cr_ / N_Li_ \| 0.02 \| 0.30 \|   IT, intratumor; IM, invasive margin; R^2^, correlation coefficient; T_tot_, total amount of T-cells, CTL, cytotoxic T-cells, TH, Helper T-cells; Treg, regulatory T-cells; N_Cr_, tumor adjacent colorectum; N_Li_, tumor adjacent liver tissue  Supplementary table 4 Clinicopathological variables and outcome   \|  \|  \| OS \|  \|  \| PFS \| \| \| \| --- \| --- \| --- \| --- \| --- \| --- \| --- \| --- \| \| Variable \|  \| HR \| 95% CI \| *P* \| HR \| 95% CI \| *P* \| \| Gender \| Male \| Ref \|  \|  \| Ref \|  \|  \| \|  \| Female \| 1.31 \| (0.6 - 2.84) \| 0.50 \| 1.12 \| (0.6 - 2.08) \| 0.73 \| \| Primary tumor \| \|  \|  \|  \|  \|  \|  \| \| Age pCRC \| <= median \| Ref \|  \|  \| NA \|  \|  \| \|  \| >median \| 2.39 \| (1.05 - 5.41) \| 0.04 \| NA \|  \|  \| \|  \|  \|  \|  \|  \|  \|  \|  \| \| TNM \| T1-T2 \| Ref \|  \|  \| Ref \|  \|  \| \|  \| T3 \| 1.47 \| (0.2 - 11.08) \| 0.71 \| 0.25 \| (0.07 - 0.87) \| 0.03 \| \|  \| T4 \| 1.64 \| (0.19 - 13.87) \| 0.65 \| 0.41 \| (0.11 - 1.56) \| 0.19 \| \|  \| N0 \| Ref \|  \|  \| Ref \|  \|  \| \|  \| N1 \| 1.80 \| (0.62 - 5.2) \| 0.28 \| 0.92 \| (0.44 - 1.94) \| 0.84 \| \|  \| N2 \| 6.84 \| (2.46 - 19.01) \| 0.00 \| 2.21 \| (1.04 - 4.69) \| 0.04 \| \|  \| M0 \| Ref \|  \|  \| Ref \|  \|  \| \|  \| M1 \| 1.16 \| (0.47 - 2.84) \| 0.75 \| 0.97 \| (0.5 - 1.87) \| 0.93 \| \| Anatomical location \| Right side \| Ref \|  \|  \| Ref \|  \|  \| \|  \| Left side/rectum \| 0.78 \| (0.33 - 1.81) \| 0.56 \| 1.02 \| (0.51 - 2.04) \| 0.95 \| \| Immunoscore \| 0-2 \| Ref \|  \|  \| Ref \|  \|  \| \|  \| 3-4 \| 1.20 \| (0.52 - 2.78) \| 0.66 \| 1.03 \| (0.41 - 2.59) \| 0.95 \| \| Colorectal liver metastases \| \|  \|  \|  \|  \|  \|  \| \| Performance status \| ECOG 0 \| Ref \|  \|  \| Ref \|  \|  \| \|  \| ECOG 1-2 \| 2.15 \| (0.97 - 4.74) \| 0.06 \| 2.33 \| (1.21 - 4.46) \| 0.01 \| \| Clinical risk score \| 0-2 \| Ref \|  \|  \| Ref \|  \|  \| \|  \| 3-5 \| 1.90 \| (0.82 - 4.38) \| 0.13 \| 1.17 \| (0.57 - 2.39) \| 0.67 \| \| Single metastasis \|  \| Ref \|  \|  \| Ref \|  \|  \| \| Multiple metastases \|  \| 1.80 \| (0.83 - 3.92) \| 0.14 \| 1.45 \| (0.76 - 2.74) \| 0.26 \| \| CEA CLM resection \| <= median \| Ref \|  \|  \| Ref \|  \|  \| \|  \| > median \| 0.67 \| (0.31 - 1.46) \| 0.31 \| 1.08 \| (0.59 - 2) \| 0.80 \| \| No NACT \|  \| Ref \|  \|  \| Ref \|  \|  \| \| NACT \|  \| 1.40 \| (0.65 - 3.04) \| 0.39 \| 1.10 \| (0.59 - 2.03) \| 0.77 \| \| Response (RECIST 1.1) \| Partial response \| Ref \|  \|  \| Ref \|  \|  \| \|  \| Stable disease \| 1.46 \| (0.49 - 4.36) \| 0.50 \| 0.99 \| (0.38 - 2.58) \| 0.98 \| \|  \| Progressive disease \| 0.40 \| (0.05 - 3.23) \| 0.39 \| 0.40 \| (0.09 - 1.8) \| 0.23 \| |
| --- | --- | --- | --- | --- | --- | --- | --- | --- | --- | --- | --- | --- | --- | --- | --- | --- | --- | --- | --- | --- | --- | --- | --- | --- | --- | --- | --- | --- | --- | --- | --- | --- | --- | --- | --- | --- | --- | --- | --- | --- | --- | --- | --- | --- | --- | --- | --- | --- | --- | --- | --- | --- | --- | --- | --- | --- | --- | --- | --- | --- | --- | --- | --- | --- | --- | --- | --- | --- | --- | --- | --- | --- | --- | --- | --- | --- | --- | --- | --- | --- | --- | --- | --- | --- | --- | --- | --- | --- | --- | --- | --- | --- | --- | --- | --- | --- | --- | --- | --- | --- | --- | --- | --- | --- | --- | --- | --- | --- | --- | --- | --- | --- | --- | --- | --- | --- | --- | --- | --- | --- | --- | --- | --- | --- | --- | --- | --- | --- | --- | --- | --- | --- | --- | --- | --- | --- | --- | --- | --- | --- | --- | --- | --- | --- | --- | --- | --- | --- | --- | --- | --- | --- | --- | --- | --- | --- | --- | --- | --- | --- | --- | --- | --- | --- | --- | --- | --- | --- | --- | --- | --- | --- | --- | --- | --- | --- | --- | --- | --- | --- | --- | --- | --- | --- | --- | --- | --- | --- | --- | --- | --- | --- | --- | --- | --- | --- | --- | --- | --- | --- | --- | --- | --- | --- | --- | --- | --- | --- | --- | --- | --- | --- | --- | --- | --- | --- | --- | --- | --- | --- | --- | --- | --- | --- | --- | --- | --- | --- | --- | --- | --- | --- | --- | --- | --- | --- | --- | --- | --- | --- | --- | --- | --- | --- | --- | --- | --- | --- | --- | --- | --- | --- | --- | --- | --- | --- | --- | --- | --- | --- | --- | --- | --- | --- | --- | --- | --- | --- | --- | --- | --- | --- | --- | --- | --- | --- | --- | --- | --- | --- | --- | --- | --- | --- | --- | --- | --- | --- | --- | --- | --- | --- | --- | --- | --- | --- | --- | --- | --- | --- | --- | --- | --- | --- | --- | --- | --- | --- | --- | --- | --- | --- | --- | --- | --- | --- | --- | --- | --- | --- | --- | --- | --- | --- | --- | --- | --- | --- | --- | --- | --- | --- | --- | --- | --- | --- | --- | --- | --- | --- | --- | --- | --- | --- | --- | --- | --- | --- | --- | --- | --- | --- | --- | --- | --- | --- | --- | --- | --- | --- | --- | --- | --- | --- | --- | --- | --- | --- | --- | --- | --- | --- | --- | --- | --- | --- | --- | --- | --- | --- | --- | --- | --- | --- | --- | --- | --- | --- | --- | --- | --- | --- | --- | --- | --- | --- | --- | --- | --- | --- | --- | --- | --- | --- | --- | --- | --- | --- | --- | --- | --- | --- | --- | --- | --- | --- | --- | --- | --- | --- | --- | --- | --- | --- | --- | --- | --- | --- | --- | --- | --- | --- | --- | --- | --- | --- | --- | --- | --- | --- | --- | --- | --- | --- | --- | --- | --- | --- | --- | --- | --- | --- | --- | --- | --- | --- | --- | --- | --- | --- | --- | --- | --- | --- | --- | --- | --- | --- | --- | --- | --- | --- | --- | --- | --- | --- | --- | --- | --- | --- | --- | --- | --- | --- | --- | --- | --- | --- | --- | --- | --- | --- | --- | --- | --- | --- | --- | --- | --- | --- | --- | --- | --- | --- | --- | --- | --- | --- | --- | --- | --- | --- | --- | --- | --- | --- | --- | --- | --- | --- | --- | --- | --- | --- | --- | --- | --- | --- | --- | --- | --- |

|  |
| --- |
|  |

OS, overall survival; PFS, progression free survival (after liver resection); HR, hazard ratio; CI, confidence interval; Ref, reference group in Cox proportional hazard analysis; pCRC, primary colorectal cancer; NA, not applicable; TNM, Tumor, node, metastases 7^th^ edition; anatomical location, right colon (coecum and 2/3^rd^ of transverse colon); ECOG, Eastern Cooperative Oncology Group performance status; NACT, neoadjuvant chemotherapy prior to liver resection
